# Epitaxial Graphene Sensors Combined with 3D-Printed Microfluidic Chip for Heavy Metals Detection

**DOI:** 10.3390/s19102393

**Published:** 2019-05-25

**Authors:** Maria Francesca Santangelo, Ivan Shtepliuk, Daniel Filippini, Donatella Puglisi, Mikhail Vagin, Rositsa Yakimova, Jens Eriksson

**Affiliations:** 1 Applied Sensors Science, Department of Physics, Chemistry, and Biology-IFM, Linköping University, S-58183 Linköping, Sweden; donatella.puglisi@liu.se (D.P.); jens.eriksson@liu.se (J.E.); 2 Semiconductor Materials, Department of Physics, Chemistry, and Biology-IFM, Linköping University, S-58183 Linköping, Sweden; ivan.shtepliuk@liu.se (I.S.); rositsa.yakimova@liu.se (R.Y.); 3 Optical Devices Laboratory, Department of Physics, Chemistry, and Biology-IFM, Linköping University, S-58183 Linköping, Sweden; daniel.filippini@liu.se; 4 Division of Physics and Electronics, Department of Science and Technology, Physics and Electronics-ITN, Linköping University, SE-58183 Linköping, Sweden; mikhail.vagin@liu.se

**Keywords:** heavy metals detection, epitaxial graphene, high sensitivity, 3D-printed flow cell, reusable lab-on-chip

## Abstract

In this work, we investigated the sensing performance of epitaxial graphene on Si-face 4H-SiC (EG/SiC) for liquid-phase detection of heavy metals (e.g., Pb and Cd), showing fast and stable response and low detection limit. The sensing platform proposed includes 3D-printed microfluidic devices, which incorporate all features required to connect and execute lab-on-chip (LOC) functions. The obtained results indicate that EG exhibits excellent sensing activity towards Pb and Cd ions. Several concentrations of Pb^2+^ solutions, ranging from 125 nM to 500 µM, were analyzed showing Langmuir correlation between signal and Pb^2+^ concentrations, good stability, and reproducibility over time. Upon the simultaneous presence of both metals, sensor response is dominated by Pb^2+^ rather than Cd^2+^ ions. To explain the sensing mechanisms and difference in adsorption behavior of Pb^2+^ and Cd^2+^ ions on EG in water-based solutions, we performed van-der-Waals (vdW)-corrected density functional theory (DFT) calculations and non-covalent interaction (NCI) analysis, extended charge decomposition analysis (ECDA), and topological analysis. We demonstrated that Pb^2+^ and Cd^2+^ ions act as electron-acceptors, enhancing hole conductivity of EG, due to charge transfer from graphene to metal ions, and Pb^2+^ ions have preferential ability to binding with graphene over cadmium. Electrochemical measurements confirmed the conductometric results, which additionally indicate that EG is more sensitive to lead than to cadmium.

## 1. Introduction

Nowadays, among water pollutants, heavy metals (HMs) are considered as the most serious source to pollute the biosphere, posing a significant threat to human health, because they are non-biodegradable and accumulate in soft tissues [1]. Some HMs are essential minerals for healthy biochemical and physiological function, since they serve as components of several key enzymes and play important roles in various oxidation-reduction reactions in human bodies. Others, such as lead, cadmium, chromium, arsenic, and mercury are toxic even when ingested in very small quantities [2]. In particular, lead, which has a high toxicity and the ability to accumulate in the body, is one of the most dangerous substances due to its negative effect on intracellular biochemical processes in living organisms [3,4,5,6,7,8,9]. Standard techniques used to detect low traces of lead are, for example, mass spectroscopy (MS) [10], inductively-coupled plasma mass spectrometry (ICPMS) [11], and atomic absorption spectroscopy (AAS) [12], which are very sensitive, accurate, and often allow for detection of different ions simultaneously, but most of them require expensive instruments and specialized staff to perform the analysis. This has led scientists to develop portable and easy-to-use methods towards real-time, accurate, and sensitive identification of HMs in the environment. Indeed, in the last few years, several sensing platforms for the detection of lead have been developed exploiting electrochemical [13,14,15,16,17,18,19,20,21,22,23,24,25,26,27,28] and conductometric detection methods [29,30], which use simple equipment and allow for the miniaturization of sensing systems. Huge progress in the development of the nano-sized materials and state-of-the-art sensing systems is well-documented in recent review papers [31]. In particular, nanomaterials-based sensors are promising in the detection of heavy metals due to their large surface area, high catalytic efficiency, high surface reactivity, and strong adsorption capacity. For all of these reasons, graphene is one of the best transducer materials because it exhibits extreme sensitivity thanks to its unique properties, such as every atom being available for interaction with adsorbing molecules [32], the high carrier mobility [33], and the high electronic conductivity even when very few charge carriers are present [34]. As a result, very small changes in epitaxial graphene (EG) conductivity can be detected leading to high-resolution sensors. Moreover, the use of nanomaterials in the design of chemical sensors has also improved their limit of detection (LoD), reproducibility, and due to the unique properties of nanoscale materials, have opened avenues for miniaturization, which has led to the emergence of lab-on-chip (LOC) technology [35].

The main trend in modern heavy metal sensors concepts has been their gradual shift from traditional electrochemical quantification of analytes to real-time non-invasive optical sensing of toxic substances, including fluorescent [36,37], surface-enhanced Raman scattering (SERS) [38], and surface plasmon resonance (SPR) sensors [39]. Nevertheless, in the present work, we investigated the performance of a sensing platform based on epitaxial graphene on Si-face 4H-SiC (EG/SiC) for liquid-phase detection of HMs, simply measuring the conductivity changes due to the interaction between Pb and/or Cd ions and the sensing surface, obtaining very promising results in terms of sensitivity and the possibility to exploit real-time monitoring. In this work, we developed and tested a reusable LOC for heavy metals detection, in which the 3D-printed microfluidic cell allowed for the interaction between the HMs solutions and the sensing surface [40]. Moreover, Density Functional Theory (DFT) calculations were performed to explain the interaction mechanisms of graphene with lead and cadmium ions, and consequentially conductivity changes of the sensing material.

## 2. Materials and Methods

### 2.1. Experimental Setup

**Figure 1:**
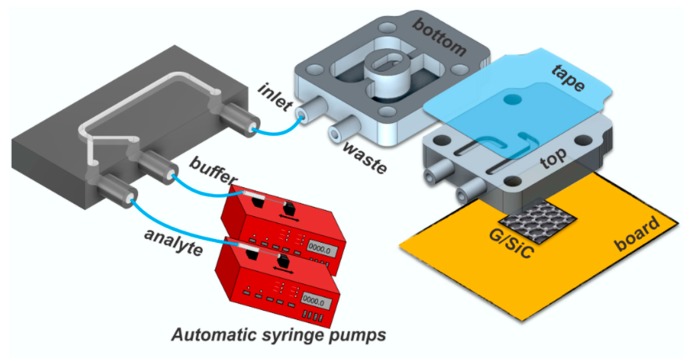
Schematic of the sensing platform.

The sensor system proposed in this work integrates the extraordinary features offered by an epitaxial graphene sensor with a 3D-printed microfluidic lab-on-chip (Figure 1). The sensor is based on a monolayer of epitaxial graphene grown on on-axis, Si-face 4H-SiC (0 0 0 1), using the well-established sublimation growth technique to produce large area, homogeneous graphene [41]. Details on the graphene growth and characteristics are reviewed in [42]. The sensor chip with a physical size of 7 × 7 mm^2^ was processed through two different sputtering steps in order to realize four circular (θ = 1 mm) electrical contacts on the corners, needed to bias the sensor and to collect the output signal. All contacts were fabricated through sputter deposition of 2 nm of titanium and 200 nm of gold sequentially [43]. Four-point measurements are possible with this scheme, but only resistance between two contacts on the diagonal of the chip was measured in this work. The microfluidic chamber, with a volume of 7 µL, was fixed on the EG surface using four screws that apply sufficient pressure to ensure that the chamber was perfectly sealed. Analyte and buffer solutions were injected using two automatic syringe pumps (NE-1010 Higher Pressure Programmable Single Syringe Pumps) with a flow-rate of 19.2 mL/h into a 3D-printed Y junction, through which the solution to send to the main chamber is selected. Buffer solution was used to clean the chip after each measurement cycle. Both chips exhibit open channels, which allow easy access for functionalization, and the sub-micro-metric surface finishing enables sealing with regular transparent adhesive tape [44]. Both microfluidic chips, which include inlet and outlet ports with an internal radius of 500 µm, were designed using Autodesk Inventor Fusion 360^®^ CAD software and printed by a Form 1 + 3D printer (FormLabs) with a proprietary resin Clear Type 02 [40]. The resin includes different proportions of modified acrylate and acrylate oligomer, epoxy monomer, acrylate monomer, photo initiator and additives as the principal components [45,46]. A 2601A Keithley Source Meter was used to bias the sensor and to collect the output signal. 

Electrochemical measurements were performed by using a computer-controlled potentiostat (Autolab, EcoChemie, Metrohm, Utrecht, The Netherlands). The custom-built electrochemical cell of O-ring type was assembled with a three-electrode system: EG/SiC, Ag/AgCl, and platinum wire were used as the working electrode, reference electrode, and counter electrode, respectively. For more information about the design of the electrochemical cell, see our previous work [47]. The stripping process was performed by square wave anodic stripping voltammetry (SWASV) at the following parameters: accumulation time of 2 min, frequency of 15 Hz, amplitude of 25 mV, and increment potential of 5 mV. Since the stripping peak current is dependent on both the redox potential of the metal and the concentration of the metal cations, the SWASV analysis enables simultaneous quantification of cadmium and lead. 

### 2.2. Sample Preparations

Analyte solutions, with concentrations of lead ions (Pb^2+^), ranging from 125 nM to 500 µM, were prepared by diluting a powder of lead chloride, PbCl_2_ (purchased from Sigma-Aldrich), in deionized water (dH_2_O). In order to compare the response of EG to other heavy metals, a concentration of cadmium ions (Cd^2+^) of 500 µM was prepared by diluting a powder of cadmium chloride, CdCl_2_ (purchased from Sigma-Aldrich), in dH_2_O. Moreover, for cross-sensitivity studies, both solutions, containing lead and cadmium ions with a concentration of 500 µM for each, were mixed and analyzed. For electrochemical measurements, aqueous solutions of Cd^2+^ and Pb^2+^ were prepared by dissolving the appropriate amounts of CdCl_2_ and Pb(NO_3_)_2_ salts in buffer solution (0.1 mol·L^−1^ HClO_4_ in Milli-Q-water) with pH = 4.5. 

### 2.3. Density Functional Theory

Since the main aim of this work is to understand the fundamental principles behind detection of the cadmium and lead in liquid phase, the experimental work is complemented by comprehensive density functional theory (DFT) calculations, with consideration of the water as a solvent phase. The nature of the interaction between heavy metals (Cd and Pb in different charge states) and graphene was elucidated using the Gaussian 09 Rev. D.01 program package [48]. As a model of graphene, C_96_H_24_ (circumcircumcoronene [49]) with edge hydrogen passivation has been chosen. All of the calculations were carried out using PBE1PBE-D3 level of theory [50] with consideration of split basis set and empirical dispersion correction (which enables us to estimate the contribution of the van-der-Waals forces into total interaction energy). The 6-31G (d) basis set was used for carbon and hydrogen atoms, while the basis set developed by the Stuttgart–Dresden–Bonn group (SDD) was utilized for the heavy metal species [51]. In order to study the solvation effect on the interaction between metal species and graphene, the self-consistent reaction field (SCRF) approach, using the polarizable continuum model (PCM) [52], was used. All atoms are enabled to be fully relaxed during the geometrical optimization procedure. All calculations were carried out without symmetry restrictions. Geometry optimization calculations were performed with SCF (self-consistent field) convergence criterion of 10^−8^. Mulliken population analysis [53] and the Hirshfeld scheme [54] were applied to study the charge distribution within interacting complexes. Since the van-der-Waals (vdW) interaction is supposed to be a prevailing factor in the adsorption of heavy metals, noncovalent interactions (NCI) analysis and topological analysis were performed using the Multiwfn program to better understand the metal–carbon (M–C) bonding [55]. The nature of M–C bonding and orbital interactions for all considered complexes were also explored by the quantum theory of atoms in molecules (QTAIM) method [56] and extended charge transfer analysis (CDA), as implemented in the Multiwfn program [57,58].

## 3. Results and Discussion

### 3.1. DFT Calculations

**Figure 2:**
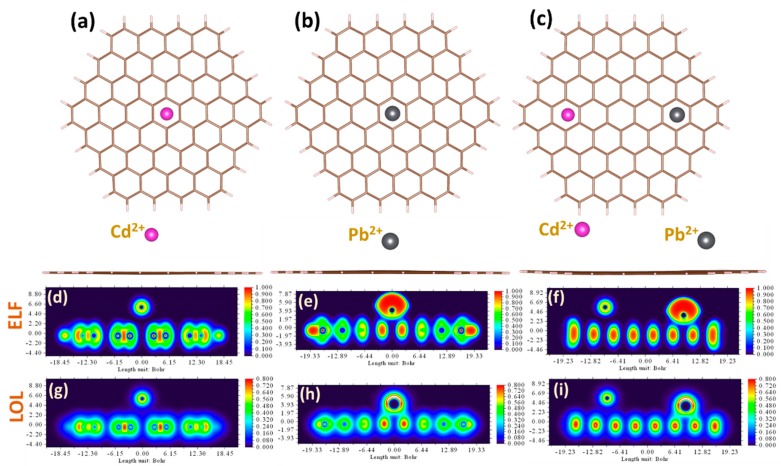
Top view and side view of the relaxed adsorption configurations of Cd^2+^ (**a**), Pb^2+^ (**b**), and simultaneous presence of Cd^2+^ and Pb^2+^ divalent ions (**c**), on the graphene surface. Contour plots of the electron localization function (ELF) and color-filled maps of localized orbital locator (LOL) for the heavy metal ions adsorbed onto graphene: Cd^2+^ on Gr (**d**,**g**), Pb^2+^ on Gr (**e**,**h**), and Cd^2+^ and Pb^2+^ on Gr (**f**,**i**), respectively.

In the experiments described in this work, the graphene response to metal-containing liquid phase has been measured for three different cases: (i) adsorption of individual cadmium ions, (ii) adsorption of individual lead ions, and (iii) simultaneous adsorption of both metals. The current work is a continuation of our previous research efforts towards deep understanding of the adsorption behavior of metal ions on graphene in aqueous phase [59]. In particular, it was revealed that the adsorption order of heavy metal ions on graphene is changed from Cd^2+^ > Pb^2+^ for gas-phase to Pb^2+^ < Cd^2+^ for water, respectively. Such reordering is originating from the solvent-mediated interaction between metal cations and carbon rings, as was explained in the classical work by Kumpf and Dougherty [60]. Furthermore, in our recent paper we demonstrated concentration dependences of the adsorption energy of lead ions and the experimentally-derived sensitivity, which may be associated with redistribution between energy components of total interaction energy. Taking the previous observations and theoretical findings into account, one can expect that the sensitivity of the graphene to Pb ions will be higher than that to Cd ions. Nevertheless, despite the theoretical predictions of binding sequence, stronger arguments are needed to better understand the way in which heavy metals interact with graphene dissolved in water electrolyte. To reach this fundamental knowledge, metal–graphene bonding is deeply investigated by comprehensive DFT calculations through performing extended charge decomposition analysis (ECDA), non-covalent interaction (NCI) analysis, and topological analysis. Prior to discussion of the nature of bonds, we focused on the adsorption configurations. As can be seen from Figure 2a–c, the most stable and favorable way for metals to be adsorbed at the graphene is for Cd^2+^ and Pb^2+^ divalent ions to occupy the hollow site (above the center of the hexagonal ring). The corresponding adsorption heights are estimated to be 3.42 Å and 2.45 Å, respectively. For each of the three cases studied, we noticed that the initial charge on ionic species (i.e., +2) before interaction with graphene tends to decrease during the interaction. This suggests that divalent metal cations on graphene act as electron-accepting adsorbates/dopants. In particular, the Mulliken/Hirshfeld charge magnitudes on separately adsorbed cadmium and lead are about +1.98/+1.90 and +1.40/+1.16, while for cadmium simultaneously adsorbed with lead on the graphene these values were slightly different for lead case: +1.98/+1.90 and +1.37/+1.12, respectively. The electron localization function (ELF) and localized orbital locators (LOL) analyses give more evidence on charge redistribution in the interacting systems (see Figure 2d–i). From the ELF and LOL images, it is clearly seen that there is no electron localization overlap between the Cd ion and graphene, while the electrons are shared between the Pb ion and carbon atoms, suggesting a larger interaction strength.

Charge decomposition analysis (CDA), which is reported in the supplementary information, revealed strong orbital interactions between the three lowest unoccupied orbitals of lead ion (LUMO, L + 1, L + 2) and the unoccupied orbitals of the graphene nanofragment. No orbital interaction between Cd^2+^ and graphene was observed.

### 3.2. Experimental Results

DFT calculations indicate that Pb^2+^ ions, adsorbed on graphene, behave as electron-accepting dopants, with a preferential charge transfer from graphene to divalent ions [61]. This process of energy transfer produces a change in the graphene conductivity, which was confirmed by our experimental measurements. Starting from PbCl_2_ diluted in dH_2_O, several concentrations of Pb^2+^ solutions, ranging from 125 nM to 500 µM, were measured. Since the water molecules on graphene act as electron-accepting (p-type) dopants [62], we observed that the presence of charged lead species in water electrolyte increased the *p*-type conductivity of graphene. 

The sensor used in this study was used for more than one year in different operating conditions (analyte concentrations, different species, etc.) without demonstrating any degradation in performance. 

**Figure 3:**
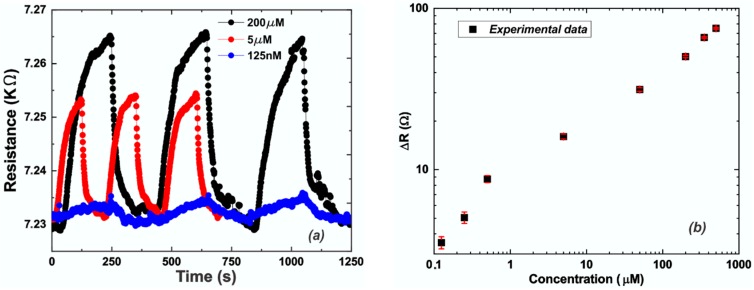
(**a**) Sensor signal (resistance) versus time for Pb^2+^ concentration of: 0.125 (blue line), 5 (red line), 200 µM (black line); (**b**) Calibration curve: experimental data (black squares) and relative error bars (red lines).

For each concentration of Pb^2+^, more than three measurement cycles were measured (signal is stable for hours, but only a few cycles are reported here) and the differential resistance (ΔR) and experimental error were calculated and are reported in Figure 3b. The resistance value reported (ΔR) is the net resistance, measured as the difference between the signal due to the presence of Pb^2+^ and the recovery value obtained by the cleaning of the chip after each measurement cycle. Figure 3a shows the EG response to 125 nM, 5 µM, and 200 µM of Pb^2+^. In particular, the response recorded for the lowest concentration of lead ions exhibits a signal-to-noise ratio (SNR) of 7.1 dB, which portends the possibility to further reduce the measurable detection limit of the sensor system.

Moreover, in accordance with Langmuir’s law which describes the adsorption of a monolayer of species onto simple surfaces, increasing the Pb^2+^ concentration leads to an increase of the ΔR value (Figure 3), since more divalent ions are adsorbed on the EG surface. DFT calculations indicated that for high levels of Pb^2+^ concentration, the energy transfer between individual lead ions and graphene decreases. It has also been confirmed by the experimental data, since we obtained higher sensitivity for low compared to high Pb^2+^ concentration, as demonstrated by the different slopes of the calibration curve reported in Figure 3b. It was observed that for low concentrations (0.125–5 µM) of Pb^2+^ we can approximate a sensitivity (S_L_ = 13.90 Ω/µM) that is much higher than the sensitivity (S_H_ = 0.10 Ω/µM) estimated for high concentrations (50–500 µM) of Pb^2+^. This result demonstrates how the system is more sensitive to low concentrations of the analyte. Moreover, a detection limit of 95 nM was extrapolated from the calibration curve (based on three times the standard deviation of the zero response, 3σ), which is lower than the recommended safe limit (180 nM) provided by the World Health Organization (WHO) for lead levels in drinking water [63]. Still, an improvement in the detection limit value is necessary to be competitive with the state-of-the-art (1–3 nM [38,39]), but this can be achieved simply by increasing the ratio between the area of the graphene surface exposed to the HMs solution and the total device area between the contacts (≈10% in this experimental configuration).

Measurements with lead dissolved in drinking water were also performed in order to evaluate the interaction between different species in complex matrixes that also includes other ions [40]. In that case, we compared the performance obtained for the highest concentration of lead dissolved in deionized and drinking water, obtaining a reduction of almost 35% of ΔR. This means that the presence of other species influences the response, and to use this system for field applications, a functionalization of the graphene surface is needed to be more selective to the different species.

**Figure 4:**
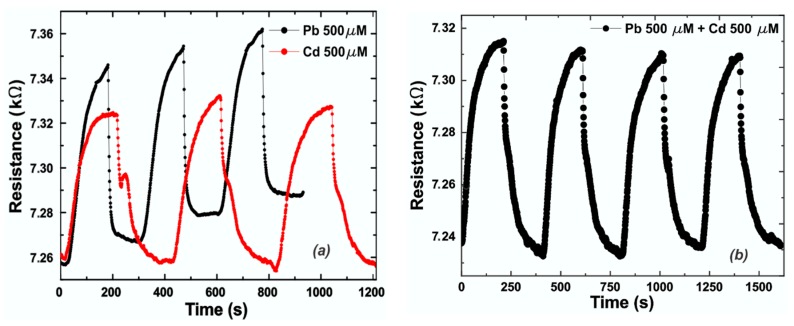
(**a**) Comparison between epitaxial graphene (EG) responses (resistance) to a concentration of 500 µM of Pb^2+^ (black line) and Cd^2+^ (red line) versus time; (**b**) EG response to a complex sample in which both solutions of Pb^2+^ and Cd^2+^ with a concentration of 500 µM were mixed.

The EG response to lead was compared to the one obtained by measuring the same molar concentration (500 µM) of cadmium ions (Cd^2+^) and is reported in Figure 4a. It was demonstrated that cadmium exhibits a lower affinity to graphene compared to lead, and it is visible from different features, like amplitude of the signal response and time response. Concerning the amplitude, in the same operating conditions in terms of molar concentrations of analyte, cadmium exhibits a response ΔR of 67 Ω compared to 76 Ω for lead (Figure 4a). Moreover, cadmium needs more time to adsorb on the graphene surface compared to lead, due to the lower affinity. The rise (t_r_) and fall (t_f_) times are the times needed to switch from 10% to 90%, or vice versa, of the signal amplitude. Both the rise and fall times of the sensor after each injection of solution containing heavy metals were analyzed. The data can be fitted assuming a decreasing exponential equation for both, in the form of:

(1) $$y=A_{0}\cdot exp\left(-t/\tau \right)+y_{0}$$

In Equation (1), *A*_0_ is a constant value depending on the reactant concentrations and whose sign depends on the filling or emptying of the reaction chamber, being positive during chamber filling (interaction metals–graphene) and negative during its emptying (cleaning-phase). *τ* is the time constant and it has different values during the filling and emptying processes. The filling time constant (*τ*_f_) is 11.2 ± 0.4 s for lead and 47.9 ± 1.2 s for cadmium. Once the data was fitted, rise (recovery time) and fall time (response time) values could be calculated, each being about three times the corresponding characteristic time (*τ_r_* and *τ_f_* respectively), hence we obtained *t_f_* = 33.6 ± 1.2 s for lead and *t_f_* = ~143.7 ± 3.6 s for cadmium.

In the worst case (highest concentration of lead), the emptying time constant (*τ*_r_) is 61.0 ± 1.4 s and consequentially, *t_r_* is 183.0 ± 4.2 s.

In both cases, the time response is calculated from a reference condition represented by the equilibrium reached when only water is flowing through the system. The time needed to reach the starting time (top of the response curve, Figure 3) is included in the recovery time.

Comparing the shape of the two signals, we observed that the signal provided by cadmium exhibits a shoulder at about half of its total amplitude, confirming the different type of interaction with the graphene surface.

To test the cross-sensitivity of the sensor system we mixed both solutions containing lead and cadmium ions with a concentration of 500 µM for each, and we observed that the resulting signal exhibits roughly the same amplitude of the lead response, an intermediate time response (around 99 s), and a little shoulder due to the presence of Cd (Figure 4b).

The presence of the shoulder is due to the different interaction nature of cadmium and graphene compared to lead, as demonstrated by DFT calculations. Moreover, due to the lower affinity with graphene exhibited by cadmium compared to lead, more time is needed for the interaction to occur. 

**Figure 5:**
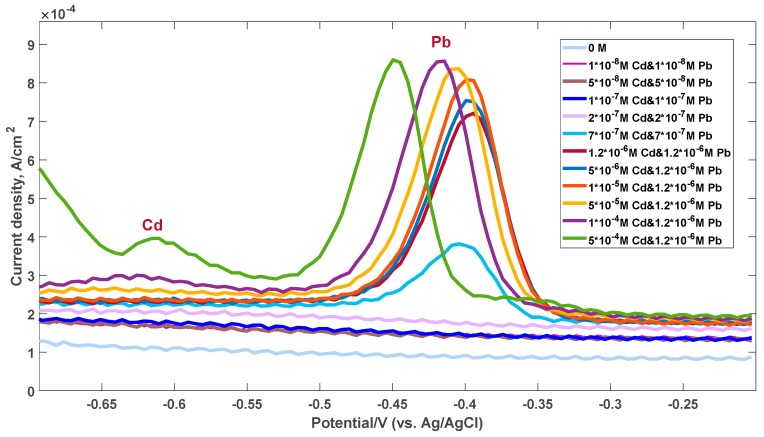
Square wave anodic stripping voltammetry (SWASV) electrochemical response of the graphene electrode for the simultaneous analysis of Cd^2+^ and Pb^2+^.

The presence of cadmium could only be detected at high concentrations (500 µM), since the EG response to lead is much stronger due to the higher affinity. This result was also confirmed by the electrochemical measurements performed and reported in Figure 5. A well-defined intensive stripping current peak (with a current density proportional to the concentration of the metal ions) is observed at approximately −0.43 V, only for the lead case. This peak starts to appear at the concentration of 500 nM Pb^2+^. The detailed description of the sensing mechanism of Pb species can be found in our previous work [47]. In contrast to detection of Pb ions, the sensitivity of the epitaxial graphene towards Cd ions is much worse. Initially, electrochemical measurements of the graphene response to exposure of both cadmium and lead ions were performed at the same metal concentrations. Since the stripping current peak is found to be very faint even at high Cd^2+^ concentrations, we then changed measurement conditions. After reaching the equilibrium concentration of 1.2 µM for both metals in solution, we then continued to increase only the Cd^2+^ concentration, while maintaining the Pb concentrations at the same level. Under accumulation conditions, the Cd-related peak was detected only at concentrations of 100 µM or higher. We noticed the enhancement of this peak at higher concentrations of metals. Such difference in behavior between the two metals can be ascribed to the interaction nature and preference for Pb adsorption compared to Cd. It is likely that under conditions of the simultaneous presence of both metals, Pb predominantly tends to occupy the available electrochemical reactive sites at the graphene surface, while Cd species are not involved in the stripping process within the corresponding concentration range. It can be explained by the very small intrinsic adsorption energy of Cd on the surface of the graphene electrode, which is not enough to accumulate the metal species required for generating the stripping current. 

**Figure 6:**
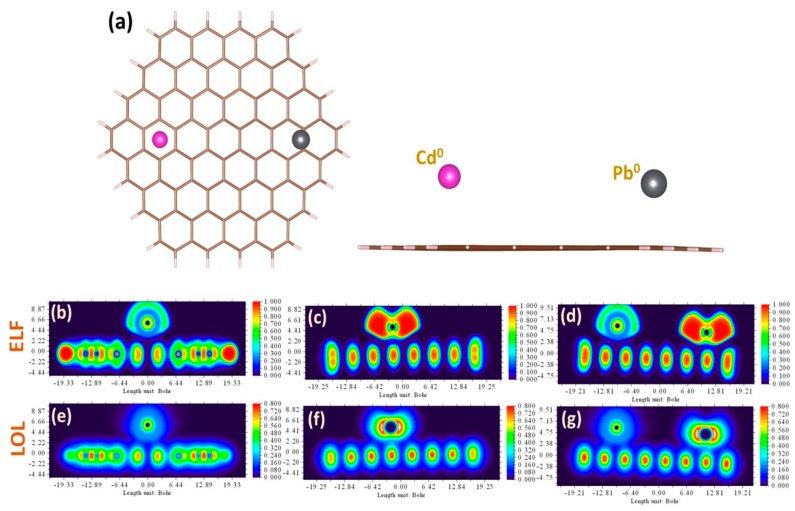
(**a**) Top view and side view of the simultaneous presence of Cd^0^ and Pb^0^ neutral metal adatoms on the graphene surface. Contour plots of the electron localization function (ELF) and color-filled maps of localized orbital locator (LOL) for the elemental heavy metal species adsorbed onto graphene: Cd^0^ on Gr (**b**,**e**), Pb^0^ on Gr (**c**,**f**), and Cd^0^ and Pb^0^ on Gr (**d**,**g**), respectively.

In most cases, at small concentrations of Cd and Pb, the corresponding stripping peaks at SWASV are well-separated. In such accumulation conditions, the intensity of the stripping peaks is mainly dependent on the binding order and adsorption preference of considered metal species on graphene. On the other hand, at high metal concentrations, the peak potential of Pb is shifted to more negative potentials as the concentration values increase, while the peak of Cd is still very weak. It means that the simultaneous quantification of Pb and Cd by graphene electrode can be attained in very limited ways and is feasible neither in a low-concentration regime due to preferential Pb adsorption, nor in the high-concentration regime due to the existence of a binary mixture of metals. To explain the experimental results, we simulate the solvent-mediated interaction between elemental heavy metals and graphene. Since the graphene response during the electrochemical process is mainly determined by the interaction between graphene and neutral Cd and Pb adatoms, we believe that a fundamental understanding of the adsorption of these heavy metals will help to address the selectivity phenomenon. In this regard, we adapted the approach, which we used to explore the adsorption of divalent ions, to investigate the behavior of elemental heavy metals in the same adsorption configurations (Figure 6a). Briefly, we revealed the Cd adatom prefers to adsorb onto the hollow site of the graphene, while Pb adsorbed on the graphene surface by interacting with two adjacent carbon atoms (so-called bridge site). Compared to Cd adsorption, Pb on graphene is the most stable adsorption configuration, with a higher adsorption energy (energy difference is higher than 0.1 eV). Charge distribution analysis of the interaction complexes by both Mulliken/Hirschfeld methods indicates that Pb is an electron-accepting dopant, with a charge magnitude of −0.051/−0.029 in individual geometry as well as −0.054/−0.036 in combined geometry, respectively. On the other hand, both methods give controversial data for charge magnitude on Cd in individual adsorption geometry (−0.012/0.064) and in combined adsorption geometry (−0.018/0.062). By analyzing the ELF and LOL plots for considered metals (see Figure 6b–g), one can conclude that the interaction between graphene and both metals is very weak and the main difference in their behavior on graphene is only related to the difference in vdW contribution to the total interaction energy. CDA analysis, which is reported in the supplementary information, also confirms the weak orbital interaction between frontier orbitals of the metal adatoms and graphene. 

## 4. Conclusions

In this work we showed design and characterization of a new sensing platform based on an epitaxial graphene sensor coupled to 3D-printed microfluidic chips for the real-time detection of heavy metals. The use of an EG sensor and a 3D-printed microfluidic chip allowed for the detection of low traces of Pb^2+^ due to the extreme sensitivity of the material. Indeed, a detection limit of 95 nM was obtained, which is much lower than the recommended limit provided by WHO for Pb levels in drinking water, and it can be still improved by increasing the EG area exposed to the solution relative to the total device area. Moreover, a full set of different concentrations of Pb^2+^ solutions, ranging from 125 nM to 500 µM, were analyzed, showing a Langmuir correlation between the signal and the Pb^2+^ concentration, fast response, good stability, and reproducibility over time. It was observed that for low-concentrations (0.125–5 µM) of Pb^2+^ the system exhibits a sensitivity (S_L_ = 13.90 Ω/µM) that is much higher than the one (S_H_ = 0.10 Ω/µM) obtained for high-concentrations (50–500 µM) of Pb^2+^. Real measurements with lead dissolved in drinking water were also performed to evaluate the interaction with different species in complex matrixes that includes other ions [40]. Comparing the performance obtained for the highest concentration of lead dissolved in deionized and drinking water, we obtained a reduction of almost 35% of ΔR. This means that the presence of other species influences the response, and to use this system for field applications, a functionalization of the graphene surface is needed to be more selective to the different species.

The comparison between the EG response to Pb and Cd was discussed, and the higher affinity to Pb was demonstrated. Indeed, from the cross-sensitivity analysis, only at high-concentration levels of Cd was it possible to detect its presence when mixed with lead. DFT calculations allowed us to get deep insights into the nature of the interaction between lead (cadmium) species and graphene as well as to explain the exceptionally high sensitivity of the EG to Pb compared to Cd. It was found that the major difference between Cd^2+^ and Pb^2+^ adsorption can be understood in terms of the charge-transfer reactions and subsequent solvent-mediated stabilization of the carbon–metal bonding. It was found that the adsorption of electron-accepting Pb ions is governed by an orbital interaction, while Cd^2+^ behavior on graphene is predominantly regulated by long-range dispersion forces. This is evidenced by NCI, ECDA, and topological analyses. The different interaction nature of cadmium and graphene compared to lead, as demonstrated by DFT calculations, introduces a small shoulder in the experimental response to Cd^2+^. Moreover, due to the lower affinity with graphene exhibited by cadmium compared to lead, more time is needed for the interaction to occur. 

To confirm the conductometric results, we have also performed electrochemical tests by the probing of simultaneously present Cd and Pb in aqueous solution using the SWASV method. Comprehensive DFT calculations enabled us to elucidate the nature of the non-bonding interaction between elemental heavy metals and graphene. It is proposed that the mechanism of preferential electrochemical detection of lead is driven by dispersion forces. 

All of the features described in this work show that this system can be used for accurate and sensitive identification of heavy metals in the environment.

## Supplementary Materials

The following are available online at https://www.mdpi.com/1424-8220/19/10/2393/s1.
