# Supplementary material for: Epitaxial Graphene Sensors Combined with 3D-Printed Microfluidic Chip for Heavy Metals Detection
Source: Sensors (Basel). 2019 May 25;19(10):2393. doi: 10.3390/s19102393 (PMC6567039; doi:10.3390/s19102393)
Supplement: Supplementary file 1 [file sensors-19-02393-s001.pdf]

Supplementary Information

Epitaxial Graphene Sensors Combined with 3D Printed Microfluidic Chip for Heavy Metals Detection

Maria Francesca Santangelo [1,*], Ivan Shtepliuk [2], Daniel Filippini [3], Donatella Puglisi [1], Mikhail Vagin[4], Rositsa Yakimova [2], and Jens Eriksson [1]

1 Applied Sensors Science, Department of Physics, Chemistry, and Biology - IFM, Linköping University, S-58183 Linköping, Sweden
2 Semiconductor Materials, Department of Physics, Chemistry, and Biology - IFM, Linköping University, S-58183 Linköping, Sweden
3 Optical Devices Laboratory, Department of Physics, Chemistry, and Biology - IFM, Linköping University, S-58183 Linköping, Sweden
4 Division of Physics and Electronics, Department of Science and Technology, Physics and Electronics - ITN, Linköping University, SE-58183 Linköping, Sweden
* Correspondence: maria.francesca.santangelo@liu.se; Tel.: +46 13 281797

DFT calculations

Charge decomposition analysis (CDA), as presented in Figure S1, revealed strong orbital interactions between the three lowest unoccupied orbitals of Lead ion (LUMO, L+1, L+2) and the unoccupied orbitals of graphene nanofragment. While no orbital interaction between Cd^{2+} and graphene is observed, as shown in Figure S1 (a, c). Even though electron-accepting Cd^{2+} dopants introduce additional energy levels in electronic structure of graphene, the frontier molecular orbitals of graphene remain unchanged after Cd^{2+} adsorption. This finding suggests that the adsorption of Cd^{2+} on graphene in the presence of water is predominantly governed by weak long-range intermolecular forces, while the bonding between Pb^{2+} and C is regulated by orbital interaction. To validate this assumption, we performed non-covalent interaction (NCI) analysis for all considered complexes. The results are illustrated in Figure S2.

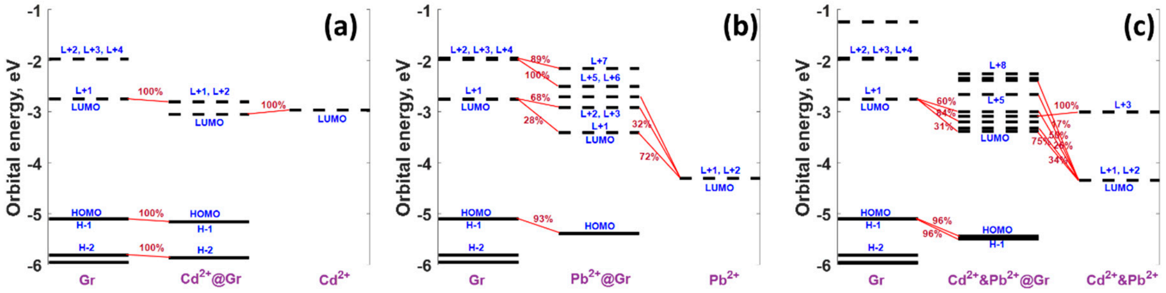

Figure S1. Orbital interaction diagrams determined using CDA analysis for three considered cases: individual Cd^{2+} on graphene (a), individual Pb^{2+} on graphene (b) and simultaneous presence of Cd^{2+} and Pb^{2+} on graphene (c). Occupied and empty molecular orbitals are represented as solid and dashed lines, respectively.

For graphene interacted with Cd^{2+}, one can distinguish three well-pronounced sharp spikes (Figure S2d), which tend to approach zero-values of reduced density gradient (RDG). The presence of two peaks within the sign(λ_2)ϱ region extending from -0.01 to 0.01 a.u. suggests an evident attractive intermolecular interaction between Cd^{2+} ion and graphene, which is dominated by weak long-range forces. This is due to the fact we consider empirical dispersion correction to mimic the adsorption of the Cd^{2+} on graphene. In the absence of this correction, no spikes within this region are observed. According to the NCI iso-surfaces at constant RDG = 0.5, this non-bonding interaction region is located at the centre of the hexagonal ring (green-coloured area in the Figure S2 a and c). It can be attributed to the presence of ring critical points (RCPs) with vanishing density

gradient. It is interesting to note that the origin of the third spike at the positive side at sign(λ_2)ϱ ≈0.02 can be interpreted as due to steric repulsion. It is important to note that this spike remains almost constant for all adsorption configurations, indicating its independency of the metal type. More complicated nature of the interaction occurs in the case of lead ions adsorption (Figure S2 e and f). More specifically, there is a competitive contribution of different components (mainly steric repulsion and strong attraction) to the total interaction energy, which is confirmed by the presence of the corresponding peaks in the NCI diagram (Figure S2 e and f) and the overlap of differently-coloured regions of the iso-surface (Figure S2 b and c). Unlike the Cd case, we did not observe the nonbonding Pb-related spikes in the vdW region. Nevertheless, the distinguishable feature related to the strong attractive interaction region is observed at the negative side, implying the negligible contribution of the dispersion forces compared to the Cd^{2+} adsorption. On the other hand, the presence of the additional spike in the repulsion region at the sign(λ_2)ϱ ≈0.027 for Pb^{2+} adsorption (Figure S2 e and f) and the absence of this spike for adsorption configuration without vdW correction (not shown here) imply the solvent-mediated van-der-Waals interaction between Pb^{2+} ion and graphene is repulsive in nature. Indeed, as was shown in our previous work [46], the vdW-corrected interaction energy between Pb^{2+} ions and graphene is much smaller than the interaction energy calculated without consideration of dispersion correction (–0.57 eV vs. –1.62 eV). Considering the results of both charge decomposition analysis and NCI analysis, it is reasonable to assume that the nature of the Pb^{2+}-C chemical bonding is mainly originating from competition between the orbital interaction and vdW repulsion.

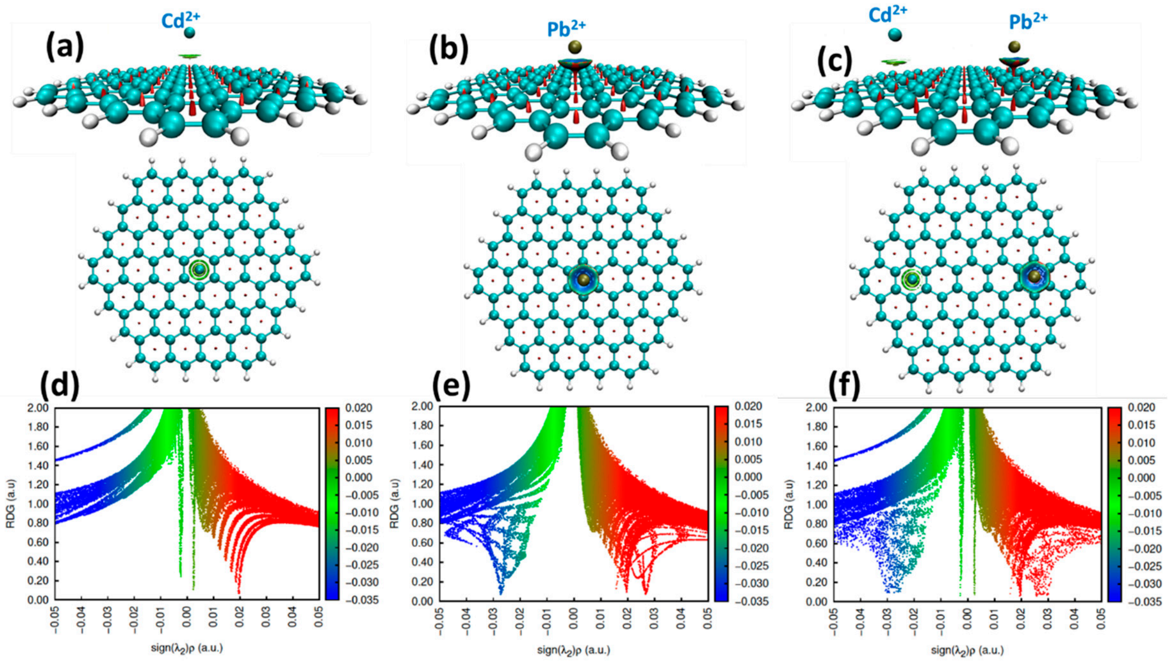

Figure S2. (side and top view) Plots of the non-covalent interaction (NCI) iso-surfaces (RDG = 0.5) for Cd^{2+} (a), Pb^{2+} (b) and Cd^{2+} and Pb^{2+} (c) adsorbed onto graphene. The iso-surfaces are colored according to sign(λ_2)ϱ over the range –0.035 to 0.02 a.u. NCI diagrams (RDG vs. sign(λ_2)ϱ) describing the interaction between graphene and Cd^{2+} (d), Pb^{2+} (e) and Cd^{2+} and Pb^{2+} (f) are depicted below. Red indicates steric repulsion region; green (light brown) indicates vdW interaction region, and blue implies the strong attractive interaction.

To shed more light on the nature of the interaction between graphene and metal cations, topological properties of critical points describing the intermolecular interaction were calculated. Topological parameters of bond critical points (BCPs), ring critical points (RCPs) and cage critical points (CCPs) are summarized in Table 1. Analysis of the data in Table 1 suggests that the electron density ϱ at BCPs is the highest for the Pb-C bonds. Laplacians of the electron densities are positive both for Cd and Pb, indicating a weak interaction with graphene in the presence of water. The relationship between potential energy density, V(r), and Lagrangian kinetic energy density, G(r), is regarded as an additional descriptor of the bond type. Data collected in Table 1 show that the -V(r)/G(r) ratio is smaller than 1 for Cd^{2+}-C bonds, whereas this ratio reaches much higher values for BCPs and RCPs describing the Pb^{2+}-C bonding. This means that the interaction between Cd with

graphene is non-bonding in nature, while Pb-C demonstrates much stronger chemical bonding (weak chemisorption). It is also evidenced by the negative value of energy density, H(r), and higher values of ELF and LOL. Thus, topological analysis also indicates that the adsorption of Cd ions on graphene is predominantly regulated by long-range dispersion forces, while the orbital interaction between unoccupied orbitals determines Pb^{2+}-C bonding. In terms of sensor applications, the above-mentioned results indicate that Pb^{2+} adsorption will cause the formation of more stable charge-transfer complexes compared to Cd^{2+} adsorption and thus, the response time of the graphene sensor to Pb^{2+}-containing water electrolyte is expected to be shorter than to Cd^{2+}-containing solution. Furthermore, since the adsorption of lead ions is a more energetically favourable process, the shape of the output signal of the graphene sensor in the simultaneous presence of Cd^{2+} and Pb^{2+} will be mainly determined by the dynamics of the Pb^{2+}-involved surface reactions at the graphene surface.

Table 1. Topological parameters for critical points (CPs), which describe the intramolecular interaction between divalent metal cations and graphene surface. Three different types of CPs, namely bond critical points (BCPs), ring critical points (RCPs), and cage critical points (CCPs) are regarded for each case.

**Topological Parameters**	**Critical points**								
	**(3, -1) BCP**			**(3, +1) RCP**			**(3, +3) CCP**		
	Cd^{2+}	*Pb^{2+}*	*Cd^{2+}/Pb^{2+}*	*Cd^{2+}*	*Pb^{2+}*	*Cd^{2+}/Pb^{2+}*	*Cd^{2+}*	*Pb^{2+}*	*Cd^{2+}/Pb^{2+}*
$\varrho(r)$	0.00273	0.0274	0.00271/ 0.0266	0.00238	0.0271	0.00252 /0.0150	0.00230	0.0156	0.00235 /0.0158
$\nabla^2\varrho$	0.0112	0.0702	0.0113/ 0.0677	0.0101	0.0740	0.0105 /0.0793	0.0103	0.0820	0.0106 /0.0831
K(r)	-0.0006	0.00126	-0.00613/ 0.00127	-0.0005	0.000441	-0.000576 /0.00103	-0.000552	-0.0033	-0.000562 /-0.00331
G(r)	0.00219	0.0121	0.00221/ 0.0117	0.00198	0.0125	0.00206 /0.0134	0.00203	0.0160	0.00209 /0.0163
ELF	0.00481	0.204	0.00464/ 0.199	0.00373	0.187	0.00415 /0.220	0.00319	0.0262	0.00322 /0.0266
LOL	0.0653	0.326	0.0642/ 0.324	0.0579	0.316	0.0609 /0.337	0.0538	0.141	0.0540 /0.141
V(r)	-0.0015	-0.0133	-0.00160/ -0.0130	-0.0014	-0.0129	-0.00148 /-0.0144	-0.00148	-0.0127	-0.00153 /-0.013
H(r)	0.00061	-0.0012	0.00613/ -0.00127	0.00056	-0.00044	0.000576 /-0.103	0.000552	0.00330	0.000562 /0.00331
-V(r)/G(r)	0.721	1.099	0.723/1.11	0.717	1.032	0.718/1.07	0.72	0.79	0.732/0.79
G(r)/ϱ(r)	0.802	0.441	0.815/0.439	0.831	0.461	0.817/0.893	0.882	1.025	0.88/1.031

Moreover, CDA analyses for three considered cases: individual Cd^0 on graphene (a), individual Pb^{2+} on graphene (b), and simultaneous presence of Cd^0 and Pb^0 on graphene (c), confirm the weak orbital interaction between frontier orbitals of the metal adatoms and graphene (Figure S3). One can observe only the orbital interaction between L+1, L+2 orbitals belonging to Pb and L+1, L+2, L+3 orbitals of the graphene. The careful analysis of NCI diagrams and iso-surfaces (see Figure S3) as well as topological analysis (Table 2) also confirm the non-bonding nature of the solvent-mediated interaction between graphene and elemental heavy metals, thereby indicating the dominant role of the vdW forces and showing the higher preference for Pb adsorption. This is evidenced by the presence only of the metal-related spikes within the vdW regions of the corresponding NCI diagrams (Figure S4) and by higher values of topological parameters describing the existing critical points.

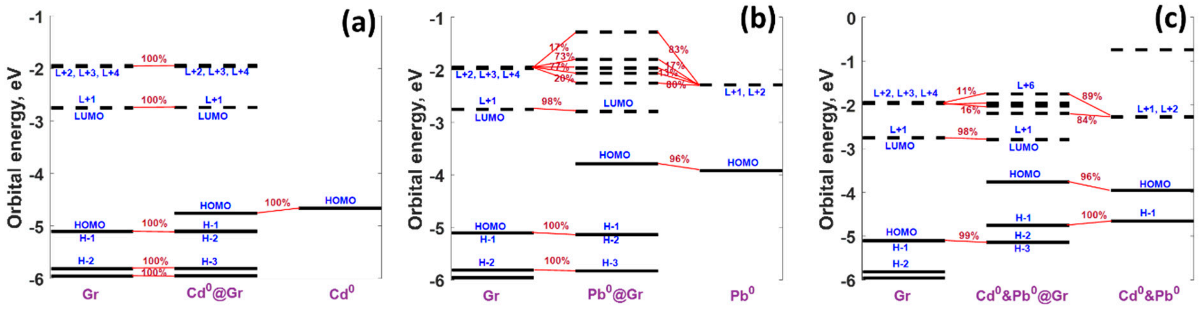

Figure S3. Orbital interaction diagrams determined using CDA analysis for three considered cases: individual Cd^0 on graphene (a), individual Pb^{2+} on graphene (b), and simultaneous presence of Cd^0 and Pb^0 on graphene (c). Occupied and empty molecular orbitals are represented as solid and dashed lines, respectively.

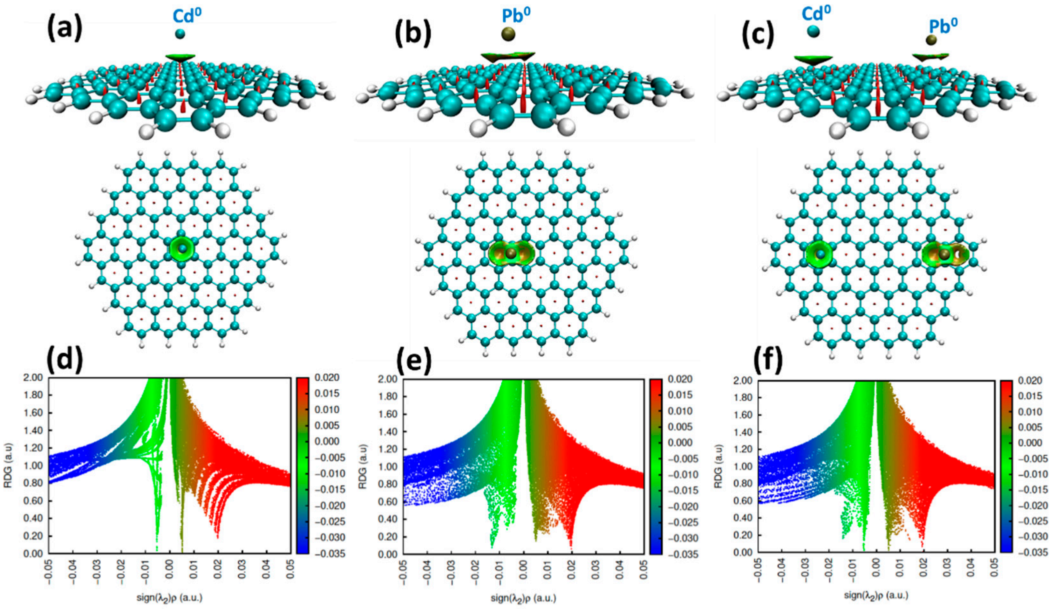

Figure S4. (side and top view) Plots of the non-covalent interaction (NCI) iso-surfaces (RDG = 0.5) for Cd^0 (a), Pb^0 (b), and Cd^0 & Pb^0 (c), adsorbed onto graphene. The iso-surfaces are colored according to sign(λ_2)ϱ over the range –0.035 to 0.02 a.u. NCI diagrams (RDG vs. sign(λ_2)ϱ) describing the interaction between graphene and Cd^0 (d), Pb^0 (e), and Cd^0 & Pb^0 (f), are depicted below. Red indicates steric repulsion region; green (light brown) indicates vdW interaction region, and blue implies the strong attractive interaction.

Table 2. Topological parameters for critical points (CPs), which describe the intramolecular interaction between elemental metal species in the neutral charge state and graphene surface. Three different types of CPs, namely bond critical points (BCPs), ring critical points (RCPs), and cage critical points (CCPs) are considered for each case.

**Topological Parameters**	**Critical points**								
	**(3,-1) BCP**			**(3,+1) RCP**			**(3,+3) CCP**		
	Cd^0	Pb^0	Cd^0/Pb^0	Cd^0	Pb^0	Cd^0/Pb^0	Cd^0	Pb^0	Cd^0/Pb^0
ϱ(r)	0.00526	0.0134	0.00519/0.014	0.00527	-	0.00519/-	0.0045	-	0.00447/-
∇^2ϱ	0.0114	0.0382	0.0111/0.0379	0.0114	-	0.0111/-	0.0130	-	0.0131/-
K(r)	-0.000326	-0.000621	-0.000311/-0.00053	-0.00032	-	-0.000314/-	-0.000385	-	-0.000389/-
G(r)	0.00252	0.0064	0.00247/0.00638	0.00253	-	0.00248/-	0.00287	-	0.00289/-
ELF	0.0316	0.0826	0.0313/0.0945	0.0315	-	0.0312/-	0.0147	-	0.0142/-
LOL	0.153	0.227	0.152/ 0.240	0.153	-	0.152/-	0.109	-	0.107/-
V(r)	-0.00219	-0.00578	-0.00216/-0.00585	-0.0022	-	-0.00216/-	-0.00248	-	-0.0025/-
H(r)	0.000326	0.000621	0.000311/0.000534	0.000329	-	0.000314/-	0.000385	-	0.000389/-
-V(r)/G(r)	0.869	0.903	0.874/0.916	0.008	-	0.870 /-	0.864	-	0.865/-
G(r)/ϱ(r)	0.479	0.477	0.475/0.455	0.480	-	0.477 /-	0.637	-	0.646/-
